# Human lipocalins bind and export fatty acids through the secretory pathway of yeast cells

**DOI:** 10.3389/fmicb.2023.1309024

**Published:** 2024-01-05

**Authors:** Aslihan Ekim Kocabey, Roger Schneiter

**Affiliations:** Department of Biology, University of Fribourg, Fribourg, Switzerland

**Keywords:** apolipoprotein D, tear lipocalin, odorant binding protein, pathogen related in yeast (Pry), *Saccharomyces cerevisiae*

## Abstract

The activation of fatty acids to their acyl-CoA derivatives is a crucial step for their integration into more complex lipids or their degradation via beta-oxidation. Yeast cells employ five distinct acyl-CoA synthases to facilitate this ATP-dependent activation of acyl chains. Notably, mutant cells that are deficient in two of these fatty acid-activating (FAA) enzymes, namely, Faa1 and Faa4, do not take up free fatty acids but rather export them out of the cell. This unique fatty acid export pathway depends on small, secreted pathogenesis-related yeast proteins (Pry). In this study, we investigate whether the expression of human fatty acid-binding proteins, including Albumin, fatty acid-binding protein 4 (Fabp4), and three distinct lipocalins (ApoD, Lcn1, and Obp2a), could promote fatty acid secretion in yeast. To optimize the expression and secretion of these proteins, we systematically examined various signal sequences in both low-copy and high-copy number plasmids. Our findings reveal that directing these fatty-acid binding proteins into the secretory pathway effectively promotes fatty acid secretion from a sensitized quadruple mutant model strain (*faa1∆ faa4∆ pry1∆ pry3∆*). Furthermore, the level of fatty acid secretion exhibited a positive correlation with the efficiency of protein secretion. Importantly, the expression of all human lipid-binding proteins rescued Pry-dependent fatty acid secretion, resulting in the secretion of both long-chain saturated and unsaturated fatty acids. These results not only affirm the *in vitro* binding capabilities of lipocalins to fatty acids but also present a novel avenue for enhancing the secretion of valuable lipidic compounds. Given the growing interest in utilizing yeast as a cellular factory for producing poorly soluble compounds and the potential of lipocalins as platforms for engineering substrate-binding specificity, our model is considered as a powerful tool for promoting the secretion of high-value lipid-based molecules.

## 1 Introduction

Fatty acids (FAs) constitute the hydrocarbon chains of most lipids. As such, FAs are not only important to provide the hydrophobic portion of the membrane bilayer forming phospholipids and sphingolipids but they also serve to store metabolic energy in neutral lipids, including triacylglycerols and steryl esters. Additionally, FAs serve as crucial precursors to highly potent signaling molecules in both mammals and plants (de Carvalho and Caramujo, 2018). FAs can be synthesized intracellularly through a well-characterized fatty acid synthase complex or acquired from the extracellular environment and then need to be activated to acyl-CoAs before undergoing further metabolism. In specialized tissues such as the fat body in insects and adipose tissue in vertebrates, FAs are stored as esterified FAs within lipid droplets. During energy scarcity, these neutral lipid stores are broken down by lipases to release free FAs into circulation (Grabner et al., 2021). However, the precise mechanisms of FA secretion and the molecular components of a potential FA export pathway remain poorly understood (Thompson et al., 2010).

The best-characterized FA transporters include the bacterial FadL protein, which is responsible for transporting FAs across the outer membrane of gram-negative bacteria, and MFSD2A, a member of the major facilitator superfamily domain, which facilitates the transport of polyunsaturated FAs in the form of lysophosphatidylcholine across the blood–brain and blood–retina barriers (van den Berg et al., 2004; Chua et al., 2023). Plants export FAs from plastids into the cytosol and endoplasmic reticulum (ER) through the FA exporter, Fax1 (Li et al., 2015). In *Saccharomyces cerevisiae*, long-chain FAs can be taken up through the integral plasma membrane protein Fat1, and their uptake is thought to be coupled to acyl-CoA activation (Zou et al., 2003). While wild-type yeast cells do not secrete FAs, double mutant cells lacking the two acyl-CoA synthases, Faa1 and Faa4, fail to efficiently take up long-chain FAs and secrete FAs into the culture medium (Johnson et al., 1994; Faergeman et al., 2001; Scharnewski et al., 2008). *Faa1/4∆* double mutant cells display aberrant morphology of the ER membrane with a more dilated ER lumen and the accumulation of laminated electron-dense material (Scharnewski et al., 2008, #46464). Notably, FA secretion in *faa1/4∆* double mutant cells requires Pry1, a small ~31 kDa secreted glycoprotein that is structurally related to the plant pathogenesis-related 1 (PR1) family of proteins (Darwiche et al., 2016; Han et al., 2023). Pry1 binds to FAs and exports them through the secretory pathway, suggesting its role in facilitating FA secretion (Darwiche et al., 2017, 2018). However, this mechanism, involving stoichiometric complexes of FA bound to a secreted protein, appears energetically expensive for secreting large amount of FAs.

In this study, we investigate whether mammalian lipid-binding proteins, directed into the secretory pathway, could enhance the secretion of FAs. We selected human Albumin, along with four members of the lipocalin family of lipid-binding proteins: fatty acid binding protein 4 (Fabp4), apolipoprotein D (ApoD), tear lipocalin (Lcn1), and odorant binding protein 2A (Obp2a), to test their potential to complement the role of Pry1 and promote FA secretion. Human Albumin, a major protein in human serum, possesses multiple FA-binding sites (Curry et al., 1998; Fang et al., 2006). Fabp4, an intracellular FA-binding protein abundant in adipocytes, regulates adipogenesis and lipolysis (Esteves and Ehrlich, 2006). It is secreted into the circulation, acting as an adipokine, and is associated with metabolic diseases such as obesity and diabetes (Ertunc et al., 2015; Hotamisligil and Bernlohr, 2015; Prentice et al., 2019).

Conversely, lipocalins, although less characterized than Albumin and Fabp4, shared a common tertiary structure and secreted lipid-binding proteins known for their diverse ligand specificity (Akerstrom et al., 2000; Bishop, 2000; Flower et al., 2000; Stopková et al., 2021; Ganfornina et al., 2022; Yang et al., 2023). Lipocalins have also been used to create proteins with novel ligand specificities, known as Anticalins, and are recognized as important mammalian respiratory allergens (Virtanen, 2021; Morales-Kastresana et al., 2022). ApoD, a highly conserved lipocalin among mammals, is broadly expressed in various mammalian tissues and is associated with several diseases, such as cancer (Ren et al., 2019; Rassart et al., 2020). Lcn1 is predominantly produced by the lacrimal glands and is involved in maintaining the tear film lipid layer, exerting antimicrobial activity (Glasgow, 2021). Obp2a, a member of the odorant binding proteins, is present in various human tissues and has a large hydrophobic pocket capable of accommodating different compounds (Tegoni et al., 2000; Pelosi and Knoll, 2022). Our findings suggest that FA secretion can be augmented by the heterologous expression of mammalian lipid-binding proteins targeted into the yeast secretory pathway, offering a potential strategy to increase the production yield of hydrophobic compounds in various applications.

## 2 Results

### 2.1 A yeast strain lacking acyl-CoA synthases and lipid export proteins serves as a platform to assess the fatty acid binding and export function of human proteins

**Figure 1:**
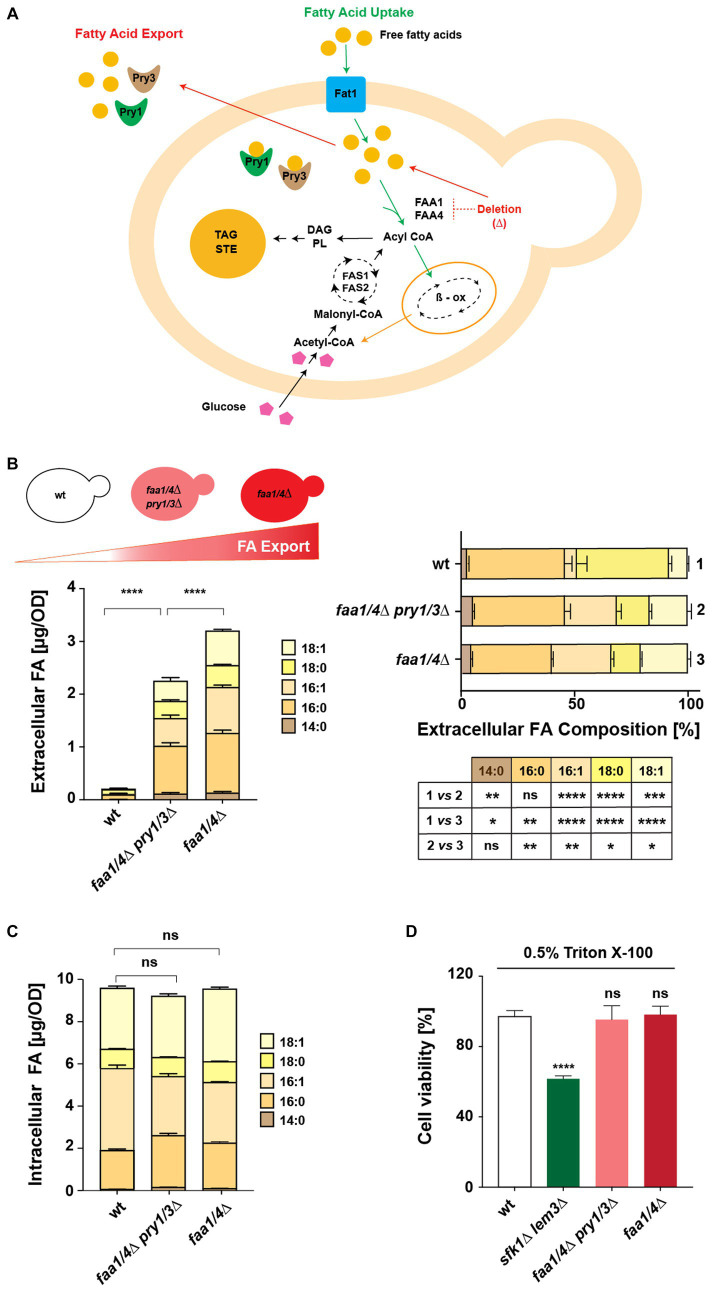
A yeast-based platform to test fatty acid-binding proteins. **(A)** Schematic illustration of the interconnections between FA synthesis, uptake, and secretion in yeast. Uptake of extracellular free FAs requires Fat1 and the acyl-CoA synthases Faa1 and Faa4. Deletion of these acyl-CoA synthases leads to FA export, facilitated by Pry proteins. Acyl-CoA, generated via synthesis or uptake, is utilized for lipid synthesis, stored as triacylglycerol (TAG) and steryl esters (STE) in lipid droplets, or channeled to peroxisomes for beta-oxidation (β-ox). **(B,C)** Comparison of intracellular and extracellular FA levels and composition in wild-type (wt), quadruple mutant *(faa1/4∆ pry1/3∆),* and double mutant *(faa1/4∆)* cells. Lipids were extracted from the cell pellet and culture supernatant, and FA methyl esters (FAMEs) were analyzed by gas chromatography. Statistical significance of changes in the composition of extracellular FAs is indicated in the table. **(D)** Viability of cells in the presence of detergents. Cells of the indicated genotypes were cultivated in YPD media with or without 0.5% Triton X-100 for 16 h at 30°C. Relative growth compared with untreated controls is shown. The *sfk1*Δ *lem3*Δ double mutant strain was included as a positive control. All data represent mean + standard deviation of at least three independent experiments. *p ≤ 0.05; **p ≤ 0.01; ***p ≤ 0.001; ****p ≤ 0.0001 (unpaired two-tailed *t*-test); ns, not significant.

A yeast strain lacking two of the six acyl-CoA synthases, Faa1 and Faa4, cannot take up exogenously supplied FAs, instead secretes free FAs into the culture medium (Johnson et al., 1994; Faergeman et al., 2001; Scharnewski et al., 2008). This export of FAs requires small soluble and secreted lipid-binding proteins of the CAP/PR1 superfamily [cysteine-rich secretory proteins (CRISP), antigen 5 (Ag5), and pathogenesis-related 1 (PR1)] (Darwiche et al., 2017; Han et al., 2023). The *S. cerevisiae* genome encodes three members of this pathogen in yeast proteins, Pry1-3 (Choudhary and Schneiter, 2012; Darwiche et al., 2018). While the deletion of any one of these three yeast *PRY* genes does not cause an apparent change in FA export, the concomitant deletion of any two of them results in a significant reduction in FA secretion (Darwiche et al., 2017) (Figure 1A). To study the effects of selected human FA-binding proteins on FA secretion, we utilized a quadruple mutant strain, lacking the two acyl-CoA synthases and both Pry1 and Pry3 (*faa1/4∆ pry1/3∆*) as a platform.

Analysis of FA levels confirmed that a wild-type strain hardly secreted any free FAs into the culture medium. Deletion of Pry1/3 led to a significant reduction of FA export compared with that of a *faa1/4∆* double mutant (Figure 1B). Notably, the composition of FAs exported by the quadruple and double mutant strains differed considerably from those secreted by the wild-type strain. The wild-type strain showed a much higher proportion of C18:0 in the culture medium compared with the strains that lacked the two acyl-CoA synthases. On the other hand, the composition of free FAs exported by the quadruple mutant (*faa1/4∆ pry1/3∆*) and the double mutant (*faa1/4∆*) was more similar, suggesting that the deletion of Pry1 and Pry3 did not strongly affect the composition of FAs that were secreted.

Intracellular FA levels and composition remained largely unaffected in both the quadruple mutant *(faa1/4∆ pry1/3∆)* and the double mutant (*faa1/4∆*) when compared with wild-type cells (Figure 1C). To examine whether the export of FAs from the *faa1/4∆* double mutant could be due to leakage from a compromised plasma membrane, we tested membrane integrity of these mutants. Both the double (*faa1/4∆*) and quadruple *(faa1/4∆ pry1/3∆)* mutants remained viable when grown in the presence of the non-ionic detergent Triton X-100 (0.5%) (Figure 1D). On the other hand, a *sfk1∆ lem3∆* double mutant, which has a known defect in plasma membrane integrity due to the deletion of the phospholipid flippase Lem3 and the regulator Sfk1, lost viability in the presence of the detergent (Mioka et al., 2018). These findings suggest that FAs are actively exported from the acyl-CoA synthase mutant cells, rather than simply leaking out through a compromised plasma membrane.

### 2.2 Addition of signal sequence to human Albumin and Fabp4 promotes their secretion

We investigated whether the expression of two abundant and secreted human FA-binding proteins, Albumin and Fabp4, could enhance FA secretion from the quadruple mutant strain. Codon-optimized versions of these genes were cloned into both low-copy and high-copy plasmids [pRS416 and pRS425, respectively (Mumberg et al., 1995)], and their expression was driven by a constitutively active alcohol dehydrogenase (*ADH1*) promoter. To target these proteins to the secretory pathway, they were fused with an N-terminal signal peptide from either yeast Pry1 (amino acids 1–19; Pry1^ss^) or pre-pro α-factor mating pheromone 1 (amino acids 1–88; MFα^ss^) (Waters et al., 1988). Previous studies aimed at optimizing the secretion of human Albumin in yeast cells had shown that the replacement of the pro-sequence (amino acids 1–6) of Albumin with that of mating factor α (MFα) increased the efficiency of secretion, and we followed a similar strategy (Sleep et al., 1990). Since Fabp4 does not have a defined signal peptide for ER luminal targeting and is secreted through an alternative pathway, we fused the signal sequences of Pry1 or that of MFα directly to the N-terminus of Fabp4 (Ertunc et al., 2015; Villeneuve et al., 2018; Josephrajan et al., 2019).

**Figure 2:**
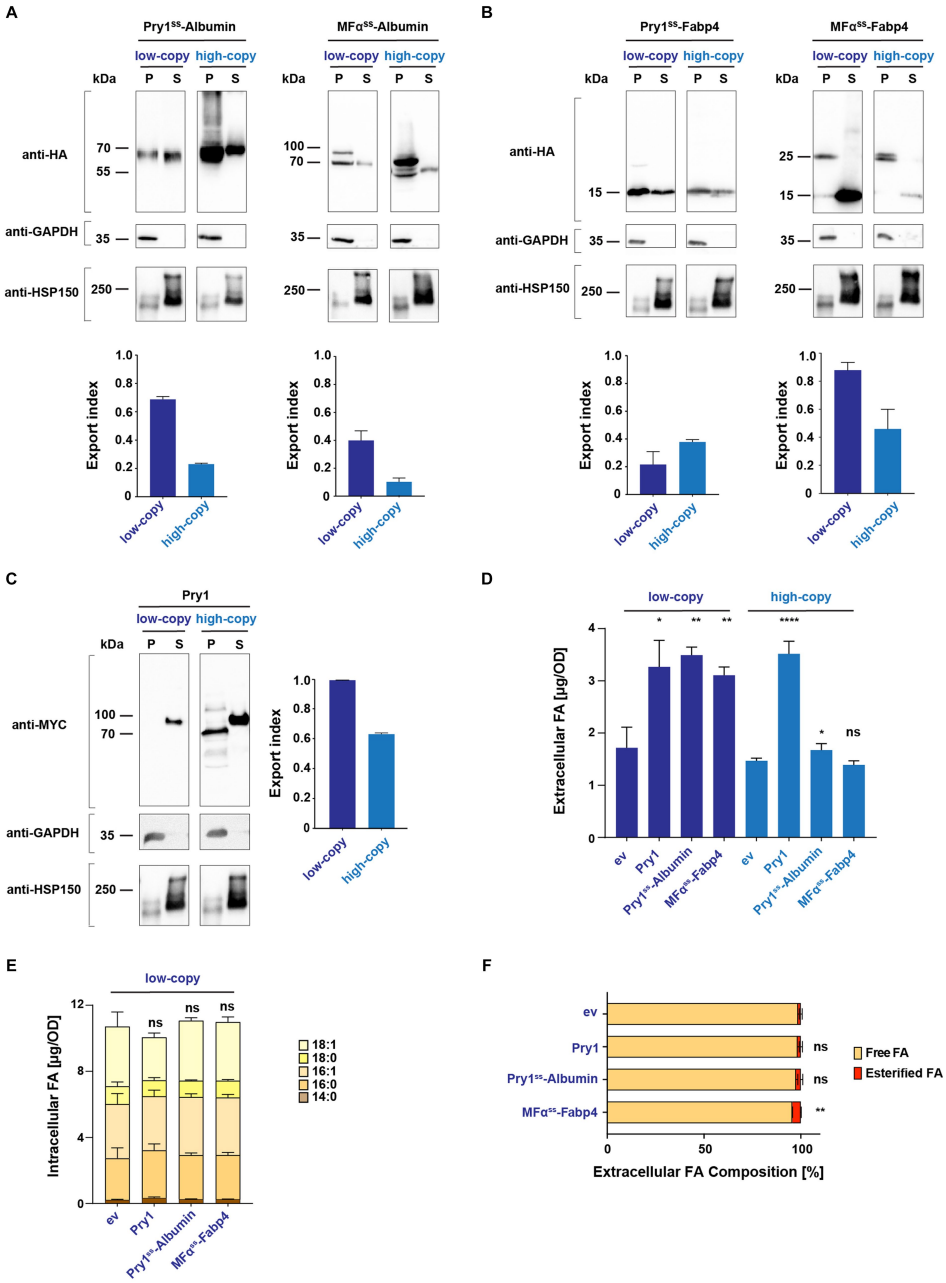
Secreted human Albumin and Fabp4 enhance fatty acid release. **(A,B)** Expression and secretion of human Albumin and Fabp4 fused to either a Pry1 signal sequence (Pry1^ss^) or the pre-pro-α-factor signal sequence (MFα^ss^), from low-copy or high-copy number plasmids. Cells were cultivated to the stationary phase, and proteins were precipitated from the cell pellet (P) and the culture supernatant (S). Proteins were separated by SDS-PAGE and probed with an antibody against the C-terminal myc-epitope of Pry1, the cytosolic glycolytic enzyme GAPDH, or a myc-tagged version of the secreted heat shock protein, Hsp150. The fraction of protein, which is secreted relative to the sum of the protein present in the intracellular and extracellular fractions, is plotted as the export index. **(C)** Secretion of endogenous Pry1 expressed from low-copy or high-copy number plasmids. Note that the higher molecular weight of Pry1 in the secreted fraction is due to protein glycosylation. The export index is plotted in the bar diagram. **(D)** FA secreted by a quadruple mutant strain *(faa1/4∆ pry1/3∆)* expressing lipid-binding proteins or an empty vector (ev) control were quantified and analyzed for their composition. **(E)** Quantification and composition of intracellular FAs of quadruple mutant strains expressing either Pry1, Pry1^ss^-Albumin, or MFα^ss^-Fabp4 from a low-copy number plasmid. **(F)** Analysis of free and esterified FAs released by control (ev, empty vector) and lipid-binding protein-expressing strains. All data represent mean + standard deviation of at least three independent experiments. Values were compared with the corresponding empty vector (ev) control by unpaired two-tailed *t*-test. *p ≤ 0.05; **p ≤ 0.01; ****p ≤ 0.0001; ns, not significant.

To examine the expression and secretion of these two human proteins in yeast, we added a hemagglutinin (HA) epitope to the C-terminus of both proteins. Western blot analysis of intracellular (cell pellet, P) and secreted proteins (culture supernatant, S) indicated that both proteins were indeed synthesized and secreted to varying degrees. To assess the efficiency of their secretion, these Western blots were quantified, and an export index, defined as the fraction of the protein present in the culture supernatant relative to the sum of the protein present in both the cell pellet and culture supernatant, was calculated and plotted in a bar diagram (Figures 2A–C). Proteins fused to the signal sequence of MFα typically displayed two discrete bands in the cell pellet. The higher molecular mass band corresponds to the immature proteins containing the MFα pre–pro signal sequence, whereas the band at lower molecular mass represents the proteolytically processed form lacking the signal sequence (Waters et al., 1988).

The results of these analyses indicated that Albumin was most efficiently (68%) secreted when expressed as a fusion with the signal sequence of Pry1 (Pry1^ss^-Albumin) from a low-copy vector (Figure 2A). Albumin has no predicted enzymatic N-glycosylation sites, but the protein is non-enzymatically glycated on lysine residues (Iberg and Flückiger, 1986; Lapolla et al., 2004). High molecular mass of possibly glycated forms of Albumin in yeast was apparent when the protein was overexpressed from a high copy number plasmids (Figure 2A).

In contrast, Fabp4 was most efficiently secreted (86%) when expressed as a fusion with the pre–pro sequence of mating factor α (MFα^ss^-Fabp4) (Figure 2B). Probing of these Western blots with antibodies against the cytosolic glycolytic enzyme glyceraldehyde 3-phosphate dehydrogenase (GAPDH) or a myc-tagged version of the secreted heat-shock protein, Hsp150, confirmed the integrity of the cell pellet and supernatant fractions (Figures 2A–C). To compare the efficiencies of secretion of Albumin and Fabp4 with that of an endogenously secreted protein, we performed the same analysis with a myc-tagged version of yeast Pry1. Pry1 reached an export index of (99%) when expressed from a low-copy plasmid, which is comparable with that of the two human proteins, indicating that they were indeed secreted efficiently (Figure 2C). Except for Pry1^ss^-Fabp4, expression of all three proteins from a high-copy number plasmid resulted in reduced secretion efficiency, possibly due to saturation of the translocation and/or ER folding machinery (Besada-Lombana and Da Silva, 2019; Lin et al., 2023).

### 2.3 Expression of both Albumin and Fabp4 promotes the secretion of fatty acids

Given that signal sequences containing Albumin and Fabp4 were indeed secreted when expressed in yeast, we next determined whether the expression of these proteins in a quadruple mutant background could increase the secretion of FAs. Therefore, levels and composition of FAs secreted by these cells into the culture medium were analyzed. Like the expression of Pry1, the expression of Albumin or that of Fabp4 significantly promoted the accumulation of FAs in the culture supernatant (Figure 2D). Expression of these proteins from a low-copy number plasmid resulted in higher levels of secreted FAs and correlated with secretion efficiency of these proteins as indicated by the export index. These findings suggest that these proteins indeed bind FAs in the ER luminal compartment and transport the lipid out of the cell through the secretory pathway. While expression of Albumin and Fabp4 promoted the accumulation of extracellular FAs, the levels and composition of intracellular FAs remained unaffected (Figure 2E). Analysis of free and esterified FAs in the culture supernatant of these strains indicated that the majority (>95) of FAs were secreted as free rather than esterified FAs (Figure 2F).

**Figure 3:**
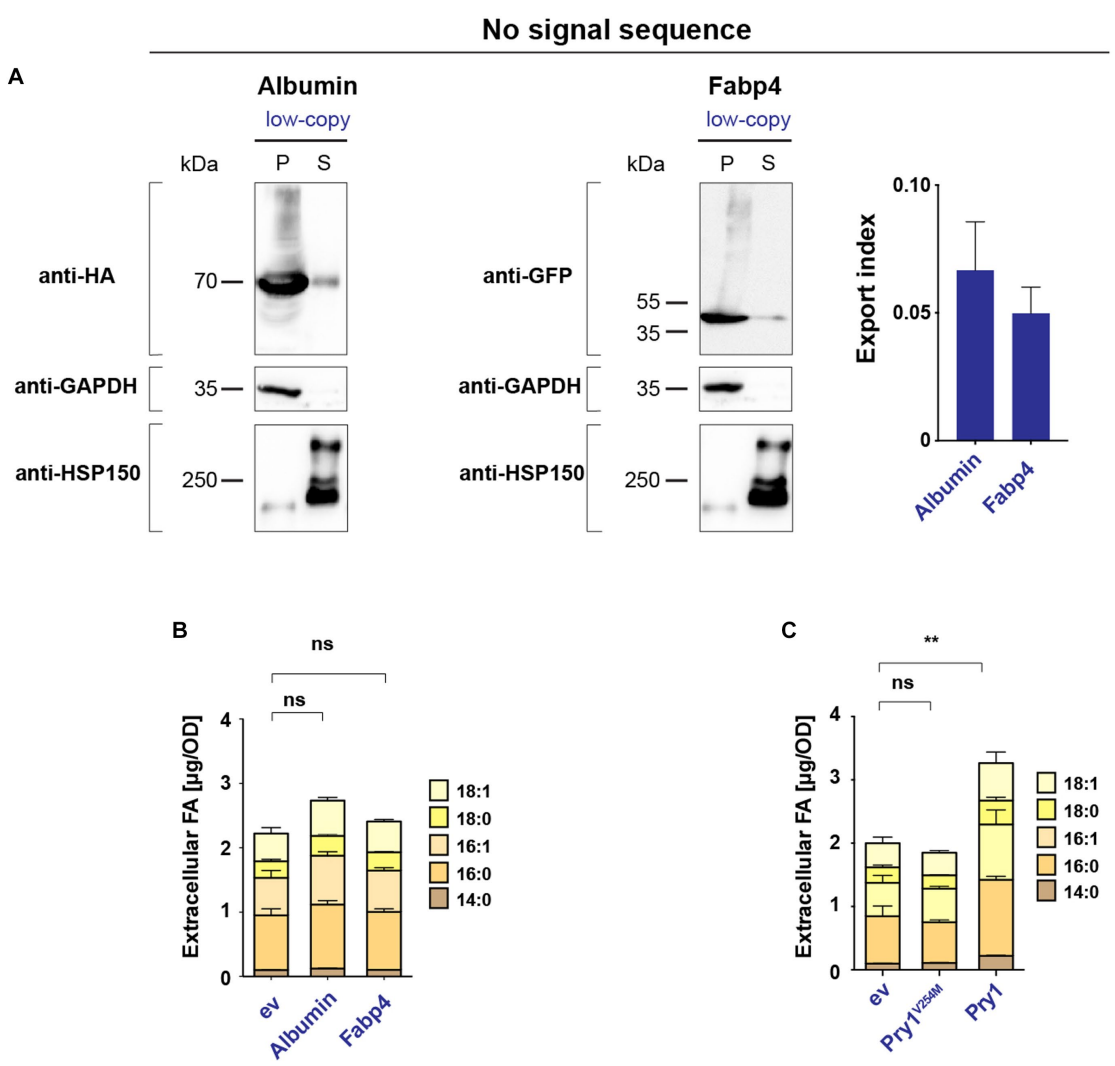
Secretion of human Albumin and Fabp4 is required to promote fatty acid export. **(A)** Western blot analysis of leader-less Albumin and Fabp4. HA- and GFP-tagged versions of Albumin and Fabp4, respectively, were expressed without a signal sequence, and their levels in the cell pellet (P) and the culture supernatant (S) were monitored by immunoblotting. Enrichment of cytosolic GAPDH and secreted Hsp150 in the respective fractions are shown. The export index is presented in the bar diagram. **(B)** Leader-less Albumin and Fabp4 do not promote FA export. Quantification of FA levels and their composition secreted by a quadruple mutant strain *(faa1/4∆ pry1/3∆)* bearing the indicated low-copy number plasmid-borne lipid-binding proteins. ev; empty vector control. **(C)** Pry1 mutant, Pry1^V254M^, expressed from a low-copy number plasmid, does not bind and does not export FAs. All data represent mean + standard deviation of at least three independent experiments. Values were compared with empty vector (ev) control by unpaired two-tailed *t*-test. **p ≤ 0.01; ns, not significant.

To confirm that FA export by human Albumin and Fabp4 indeed depends on the secretion of these proteins into the extracellular space, we generated versions of both proteins lacking a signal sequence. In the absence of a signal sequence, Albumin and Fabp4 were primarily retained in the intracellular fraction, with significantly lower levels detected in the culture supernatant (Figure 3A). When expressed in a quadruple background, these cytosolic variants of Albumin and Fabp4 did not promote FA secretion (Figure 3B). Additionally, a mutant version of Pry1, Pry1^V254M^, bearing a point mutation in the FA-binding pocket, failed to promote FA secretion when expressed in the quadruple mutant (Darwiche et al., 2017) (Figure 3C). These results indicated that the binding of FAs within the ER luminal compartment by human Albumin or Fabp4 was indeed required to promote lipid secretion and, probably as shown for Pry1, required direct binding of the FA by the transporting protein.

### 2.4 Human lipocalins, ApoD, Lcn1, and Obp2a promote the secretion of fatty acids

**Figure 4:**
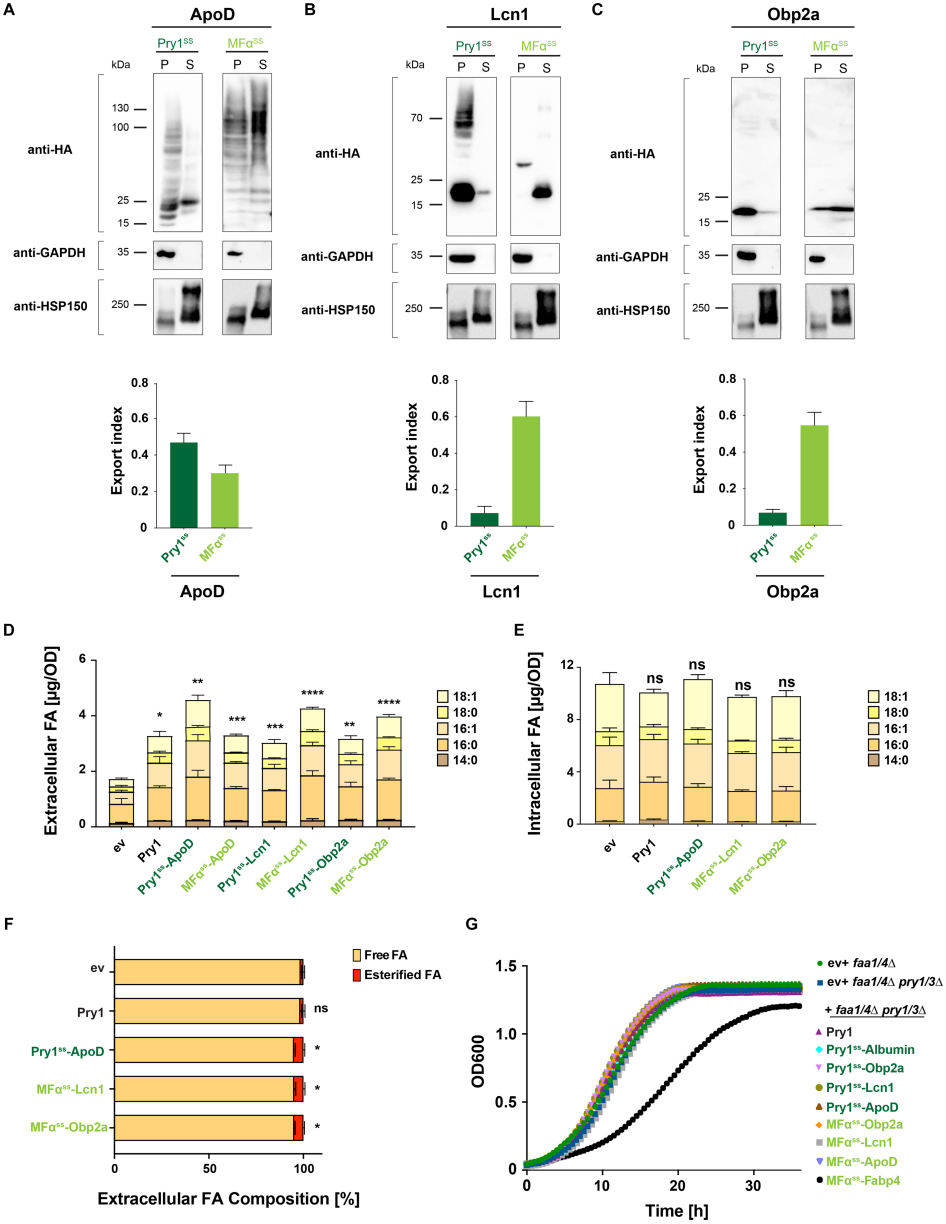
Human lipocalins promote fatty acid export. **(A–C)** Expression and secretion of human lipocalin family members ApoD, Lcn1, and Obp2a fused to either a Pry1 signal sequence (Pry1^ss^) or the pre–pro-α-factor signal sequence (MFα^ss^) from low-copy number plasmids. Cells were cultivated to the stationary phase, and proteins were precipitated from the cell pellet (P) and the corresponding volume of the culture supernatant (S). Proteins were separated by SDS-PAGE and probed with an antibody against the C-terminal HA-epitope, the cytosolic glycolytic enzyme GAPDH, or the secreted heat shock protein Hsp150-myc. Note the higher molecular weight bands observed in MFα^ss^-ApoD-expressing cells due to ER-luminal protein glycosylation. The fraction of secreted lipocalins is plotted as the export index. **(D)** Quantification of FA levels and their composition secreted by a quadruple mutant strain *(faa1/4∆ pry1/3∆)* expressing lipocalins. **(E)** Quantification and composition of intracellular FAs in quadruple mutant strains expressing specific lipocalins. **(F)** Relative levels of free and esterified FA released by control (ev) and lipid-binding protein-expressing strains. **(G)** Growth of double (*faa1/4∆*) and quadruple mutant (*faa1/4∆ pry1/3∆*) cells containing an empty vector (ev) or expressing the indicated lipid-binding protein. All data represent mean + standard deviation of at least three independent experiments. Values were compared with the corresponding empty vector (ev) control by unpaired two-tailed *t*-test: *p ≤ 0.05; **p ≤ 0.01; ***p ≤ 0.001; ****p ≤ 0.0001; ns, not significant.

We next investigated whether the expression of lipocalins could enhance FA export in yeast. We synthesized yeast codon-optimized versions of three human lipocalin family members: ApoD (amino acids 21–169), tear lipocalin Lcn1 (amino acids 19–158), and odorant binding protein Obp2a (amino acids 16–155). Their open reading frames, lacking their native mammalian signal peptide, were fused to the signal sequence of either yeast Pry1 or pre–pro-α-factor for expression from a low-copy number plasmid. All three human lipid-binding proteins were secreted to varying degrees, depending on the signal peptide used (Figures 4A–C). While Obp2a and Lcn1 were exported most efficiently when fused to MFα^ss^, ApoD was secreted best when conjugated to Pry1^ss^ (Figures 4A–C). These results indicated that the efficiency of the signal peptide and the secretion of the client protein were context-dependent (Sleep et al., 1990; Mori et al., 2015). Moreover, ApoD appeared to get highly glycosylated when fused to MFα^ss^ but not when expressed with the signal sequence from Pry1 (Figure 4A). Previous studies have shown that ApoD harbors two *N*-glycosylation sites, Asn-65 and Asn-98, and their glycosylation might impact lipid binding and FA secretion (Subramanian and Gundry, 2022).

Analyses of FA exported by a quadruple mutant strain expressing these lipocalins indicated that all three of them significantly promoted FA export (Figure 4D). The level of FA that was exported correlated with the secretion efficiency of the respective lipocalin (Figures 4A–D). The levels and composition of intracellular FAs of strains, secreting high levels of FAs, were not significantly altered by the expression of any of the three lipocalins (Figure 4E). Notably, the majority (>94%) of these FAs were secreted as free rather than esterified FAs (Figure 4F). All three lipocalin expressing strains showed a low but significant increase in the export of esterified fatty acids. This could possibly be explained by an enhanced export of fatty acid ethyl esters, which are formed in strains lacking acyl-CoA synthases (Scharnewski et al., 2008). Expression of FA-binding proteins in the quadruple mutant background (*faa1/4∆ pry1/3∆*) did not significantly affect the growth rate of these cells. Except for cells expressing Fabp4, cells grew comparable to double (*faa1/4∆*) or quadruple mutant (*faa1/4∆ pry1/3∆*) cells (Figure 4G), suggesting that the expression of these human FA-binding proteins does not impair the fitness of these strains under the conditions tested.

### 2.5 ApoD efficiently exports fatty acids and shows binding preference for unsaturated long-chain fatty acids

**Figure 5:**
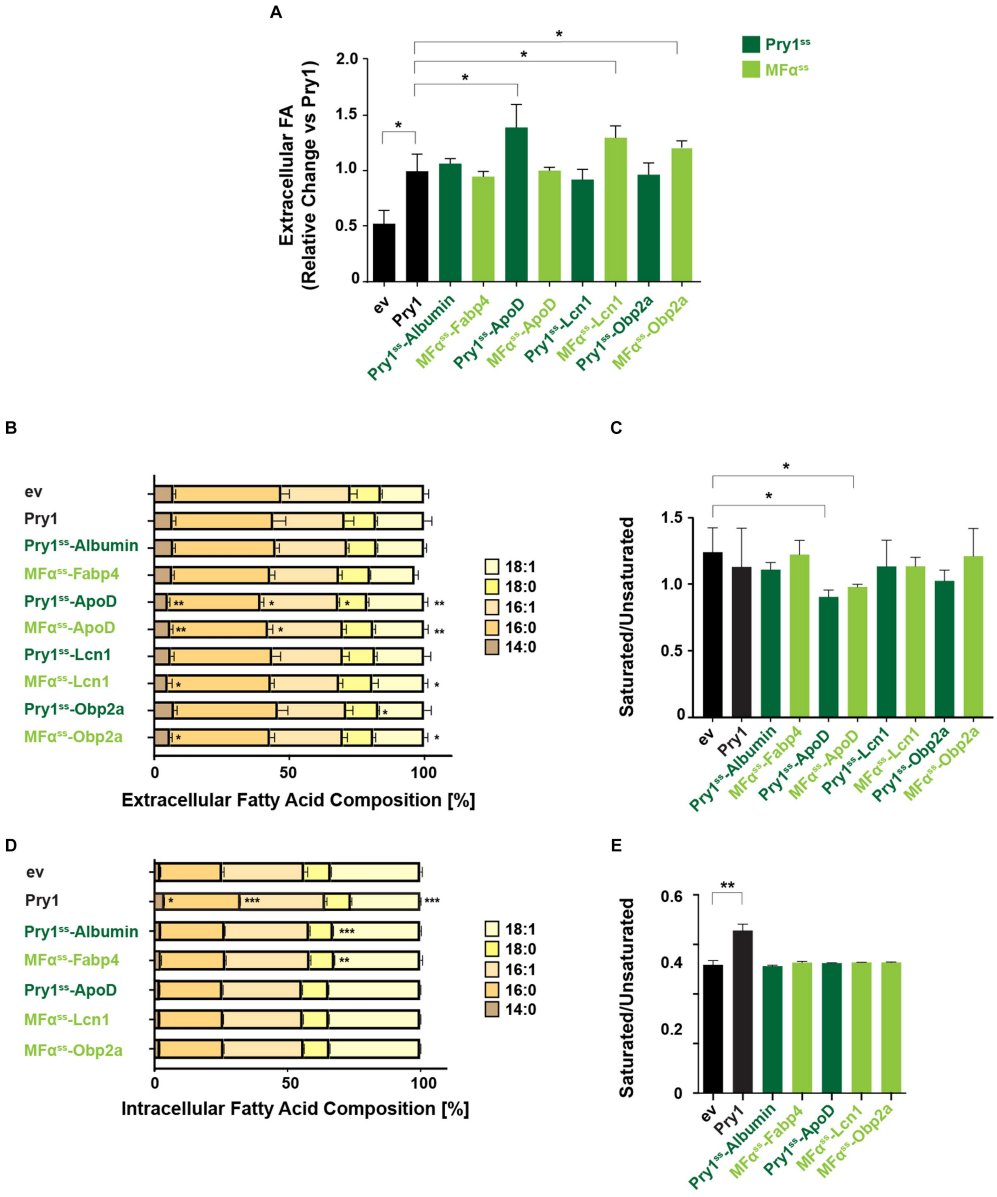
ApoD shows preference for exporting unsaturated fatty acids. **(A)** Fold change of total extracellular FA of human lipid-binding protein expressing strains relative to the Pry1 expressing strain. Significant deviations compared with Pry1-expressing strains are indicated. *p ≤ 0.05 (unpaired two-tailed *t*-test). **(B,D)** Analysis of relative FA composition in the extracellular **(B)** or intracellular **(D)** FA pools of quadruple mutant strains *(faa1/4∆ pry1/3∆)* expressing the indicated lipid-binding proteins. **(C,E)** The ratio of saturated to unsaturated FAs of the extracellular **(C)** and intracellular **(E)** FA pools is shown. All data represent mean + standard deviation of at least three independent experiments. Significant deviations compared with control cells bearing an empty vector (ev) are indicated. *p ≤ 0.05; **p ≤ 0.01; ***p ≤ 0.001 (unpaired two-tailed *t*-test). Lipid-binding proteins expressed with a Pry1^ss^ signal sequence are shown in dark green; those expressed with a mating factor alpha signal sequence (MFα^ss^) are indicated in light green.

To examine whether lipocalins are more efficient in exporting FAs than Pry1, we normalized FA secretion of lipocalin-expressing cells to that of Pry1. This analysis revealed that all three lipocalins, ApoD, Lcn1, and Obp2a, secreted higher levels of FA than did the Pry1 expressing quadruple mutant strain (Figure 5A). To determine whether expression of these human lipid-binding proteins affected the relative composition of intracellular or extracellular FAs, we compared the normalized composition of extracellular and intracellular FAs (Figures 5B,D). The relative composition of the extracellular FA pool was slightly but significantly affected cells expressing ApoD, Lcn1, or Obp2a (Figure 5B). The ratio between saturated and unsaturated FAs present in the extracellular pool was varying between 0.8 and 1.4 and thus significantly higher than that of the intracellular pool, which is approximately 0.5 (Figures 5C,E). Interestingly the ratio between saturated to unsaturated FA was significantly lower in cells expressing ApoD, indicating that the protein preferentially secreted unsaturated FAs (Figure 5C). Given that the intracellular FA composition of the ApoD expressing strain was not shifted toward more unsaturated fatty acids, this observation suggest that ApoD has preference toward binding unsaturated FAs. This is consistent with the reports showing that ApoD binds to polyunsaturated n-6 FAs, such as arachidonic acid (Morais Cabral et al., 1995).

In addition, cells expressing Albumin or Fabp4 displayed a small but significant decrease of stearic acid, C18:0, in their intracellular FA pool, without affecting the overall ratio between saturated and unsaturated FAs (Figures 5D,E). On the other hand, cells expressing Pry1 showed elevated intracellular levels of saturated FAs, and this affected the ratio between saturated and unsaturated FAs (Figures 5D,E). This is in line with the observation that Pry1 preferentially binds to saturated fatty acids *in vitro* (Darwiche et al., 2017).

In summary, our study demonstrates that human lipocalins efficiently bind and promote FA export when expressed in yeast cells, lacking key enzymes needed for FA activation and export. These findings shed light on the potential of utilizing human lipocalins to modulate FA export and may have implications in various biotechnological and biomedical applications.

## 3 Discussion

Understanding the mechanisms governing FA export is crucial, given its relevance to metabolic disorders, such as diabetes, obesity, and chronic inflammation (Glatz et al., 2010). Additionally, there is increasing interest in manipulating metabolic pathways to induce the efflux of FA-derived products for biofuel production (Sheng and Feng, 2015; Teixeira et al., 2017). Building on our prior identification of the function of Pry proteins in FA-binding and export, we aimed to explore this protein-mediated FA transport and its potential applications in yeast (Darwiche et al., 2017). In this study, we developed a yeast model to investigate human lipid-binding proteins, their role in FA transport, and their potential use in secreting hydrophobic membrane-impermeable compounds.

The essentiality of Pry proteins in yeast for the viability of a *faa1/4∆* double mutant, which secretes FAs, and the direct binding of Pry1 to free FAs *in vitro*, suggest that these CAP family members play a critical role in shuttling FAs through the secretory pathway out of cells (Darwiche et al., 2017). Moreover, previous observations indicate that free FAs do not permeate the plasma membrane passively; rather, they require an active transport system for efflux (Kamp and Hamilton, 2006; Kampf and Kleinfeld, 2007). While yeast cells use Pry family members for this active transport, the mechanism of FA release from dedicated animal tissues, such as adipocytes, remains less clear. Evidence suggests that FA efflux from adipocytes is protein mediated, as it can be inhibited by DIDS (4,4′-diisothiocyanatostilbene-2,2′-disulfonic acid), an inhibitor of ABC transporters and anion exchangers (Henkin et al., 2012). However, the exact nature of this pathway and its components await further molecular characterization. Recent studies also implicate integral membrane proteins such as Solute Carrier Slc43a3 in regulating the flux of free FAs in adipocytes (Hasbargen et al., 2020). These types of FA transport systems would likely require additional factors for the extraction of the free FA from the exoplasmic leaflet of the plasma membrane. Our findings demonstrate that the expression and targeting of human lipid-binding proteins to the secretory pathway of a model yeast is a valuable approach to test the FA-binding properties of candidate proteins *in vivo* and suggests that proteins such as Albumin or Fabp4 have the potential to serve as active FA transporters.

From a broader perspective, metabolites are often unable to passively permeate membranes, leading to their intracellular accumulation, potential toxicity, or feedback inhibition of their own production. Developing transport pathways to facilitate the secretion of such compounds from cells can mitigate these challenges and enhance their yield by providing a metabolic sink. For example, ubiquinone production can be improved by the expression of the human lipid-binding/transfer protein saposin B, which extracts the membrane-bound ubiquinone and makes the saposin-bound lipid water-soluble (Xu et al., 2016).

A metabolite trafficking approach similar to the one we employed for FAs was recently used to enable the secretion of membrane-impermeable terpenes in yeast (Son et al., 2022). This involved fusing a terpene-binding protein, the lipid-binding domain of supernatant protein factor (SPF), with a signal peptide for the selective secretion of squalene and β-carotene (Son et al., 2022). Combining enhanced export pathways with improved fluxes through specific pathways, as demonstrated in the mevalonate pathway, could further boost compound yields and simplify their recovery (Li et al., 2022; Lu et al., 2022). Yeast is considered as an excellent platform for developing microbial factories to produce high-value compounds (Payen and Thompson, 2019). Notably, efforts to enhance the production of FA-derived oleochemicals and biofuels through bioengineering of synthesis, storage, and degradation pathways have been successful (Zhou et al., 2016; Ferreira et al., 2018; Yu et al., 2018; Yan and Pfleger, 2019; Liu et al., 2022). Overexpression of diacylglycerol acyltransferase *DGA1*, along with the triacylglycerol lipase *TGL3*, in a mutant background lacking acyl-CoA synthases and peroxisomal enzymes, yielded high levels of extracellular free FAs (2.2 g/L) (Leber et al., 2015). In comparison, the *faa1/4∆* double mutant employed here generated an approximately 100-fold lower titer of FAs (Scharnewski et al., 2008). However, this titer is high enough to test the function of lipid-binding proteins in promoting FA secretion.

Our study shows that targeting FA-binding proteins to the secretory pathway of a yeast platform holds promise for characterizing the *in vivo* ligand specificity of human lipid-binding proteins. Given the diverse ligands known to bind lipocalins and ongoing engineering efforts to tailor them for specific substrates, these proteins present an attractive toolbox to optimize the secretion of specific lipids, lacking a naturally occurring binding protein.

## 4 Materials and methods

### 4.1 Yeast strains and growth conditions

*S. cerevisiae* strains deleted for multiple genes were generated using PCR deletion cassettes and marker rescue strategies as described before (Darwiche et al., 2017). Strains were grown in a minimal defined medium (containing 0.67% yeast nitrogen base without amino acids (US Biological, Salem, MA, United States), 0.73 g/L of an amino acid/nucleobase mixture, and 2% glucose) at 30°C under shaking condition at 180 rpm. The amino acid/nucleobase mixture contained 1.0 g adenine, 1.0 g argenine, 1.0 g histidine, 3.0 g leucine, 11.5 g lysine, 1.0 g methionine, 15.0 g threonine, 1.0 g tryptophan, and 2.0 g uracil (all from AppliChem GmbH, Darmstadt, Germany). For fatty acid analyses, pre-cultures were grown overnight in either SC-Ura or SC-Leu medium. The next morning, the main cultures were inoculated at 0.1 OD_600nm_ and after 24 h of growth, 3 or 5 OD_600nm_ units of cells and the corresponding culture medium were collected.

### 4.2 Generation of strains expressing human lipid-binding proteins

Yeast codon optimized cDNA-encoding human Albumin, Fabp4, Obp2a, Lcn1, and ApoD, which were synthesized (GenScript, Piscataway, NJ) (sequences are available in Supplementary material). Genes were amplified by PCR and cloned by homologous recombination into BamH1/SalI-digested low-copy and high-copy number plasmids [pRS416 and pRS425, respectively (Mumberg et al., 1995)], driving their expression from a constitutive alcohol dehydrogenase promoter (*ADH1*) and as fusion proteins with signal sequences derived either from pre–pro-mating factor alpha (MFα^ss^, amino acids 1–88) or Pry1 (Pry1^ss^, 1–19) (Waters et al., 1988; Choudhary and Schneiter, 2012). Transcription was terminated by the inclusion of cytochrome C, isoform 1, terminator sequence (*CYC1*) (Mumberg et al., 1995). PCR-amplified cDNA fragments of human Obp2a, Lcn1, and ApoD and yeast Pry1 were cloned into low-copy number plasmids, pRS416-ADH-MFα^ss^ and pRS416-ADH-Pry1^ss^, which were digested with HindIII/EcoRI and NheI, respectively. Plasmids were transformed into yeast strains, and transformants were selected on media lacking either leucine or uracil (Gietz and Woods, 2002). All plasmids were verified by sequencing.

### 4.3 Analysis of protein secretion and Western blotting

To assess the expression and secretion of proteins of interest, they were C-terminus-tagged with either hemagglutinin (HA), a myc-epitope, or GFP. For Western blotting, cellular proteins were extracted from 3 OD_600nm_ units of cells with 185 mM NaOH and precipitated with 10% trichloroacetic acid (TCA) (Horvath and Riezman, 1994). To assess their secretion into the culture medium, proteins from the culture medium were precipitated with 10% TCA; the pellet was washed with acetone and solubilized in a loading buffer for further analysis by SDS-PAGE and Western blotting. After blotting, nylon membranes were probed using rat anti-HA antibody (Roche #11867423001, 1:2,000), a mouse monoclonal antibody against GFP (Roche #11814460001, 1:2,000), anti-MYC (Invitrogen #13–2500, 1:5,000), or anti-GAPDH (1:5,000). Secondary antibodies such as horseradish peroxidase (HRP)-conjugated goat anti-rat (Merck #AP136P, 1:10,000) or goat anti-mouse (Bio-Rad #1706516, 1:10,000) were used. A chemiluminescence reaction was developed using the Immobilon Forte Western HRP substrate (Merck), and the signal was recorded with an ImageQuant LAS 4000 biomolecular imager (GE Healthcare). Signals were quantified by pixel analysis using ImageJ (Fiji), and the export index was calculated as the ratio of secreted protein relative to the sum of proteins in the intracellular and the secreted fractions (Schindelin et al., 2012).

### 4.4 Total fatty acid analysis

For the analysis of total extracellular FAs, the culture medium from 3 or 5 OD_600nm_ units of cells was collected, cells were removed by two successive steps of centrifugation (1,500 × g for 3 min and 13,200 × g for 3 min, respectively), and the medium was dried completely in a lyophilizer. For intracellular FAs, 5 OD_600nm_ units of cells were collected, cells were washed two times with H_2_O, and the cell pellet was dried in a lyophilizer. FA methyl esters (FAMEs) were generated by incubating the dried fraction in methanol-sulfuric acid (5%; v/v), supplemented with butylated hydroxytoluene (0.01%; w/v) and an internal standard (C17:0) at 85°C for 45 min. FAMEs were extracted with organic solvents (hexane/NaCl (0.9% w/v); 2:1, v/v), dried, and resuspended in heptane. FAMEs were separated on an Agilent 7890A gas chromatograph (GC) equipped with a DB-23 capillary column (30 m × 0.25 mm × 0.25 μm) (Agilent Technologies, Santa Clara, CA) and quantified relative to an internal standard (C17:0, 10 μg) as described before (Darwiche et al., 2017).

### 4.5 Free fatty acid analysis

To determine the levels of free FAs in the extracellular fraction, the culture medium from 6 OD_600nm_ units of cells was collected, cells were removed by two successive steps of centrifugation, and lipids were extracted in chloroform:methanol:HCl (50:100:1.5; *v/v*), supplemented with an internal standard (C17:0). The organic phase was collected and dried under a stream of nitrogen. Dried lipids were resuspended in 200 μL of methanol and divided into two aliquots for the analysis of both free and total FAs. To quantify free FAs, lipid extracts were methylated in methanol with 1-ethyl-3-(3-dimethylaminopropylcarbodiimide). Methylated free FAs were extracted with organic solvents [hexane/Tris–HCl (0.1 M, pH 7.5) (5:1, *v/v*)], and lipids were then dried, resuspended in heptane, and analyzed as described for FAMEs.

## Data availability statement

The original contributions presented in the study are included in the article/Supplementary material, further inquiries can be directed to the corresponding author.

## Author contributions

AE: Conceptualization, Data curation, Formal analysis, Investigation, Methodology, Visualization, Writing – original draft. RS: Conceptualization, Formal analysis, Funding acquisition, Investigation, Methodology, Project administration, Resources, Supervision, Validation, Visualization, Writing – review & editing.

## References

[ref1] Akerstrom B. Flower D. R. Salier J. P. (2000). Lipocalins: unity in diversity. Biochim. Biophys. Acta 1482, 1–8. doi: 10.1016/s0167-4838(00)00137-0, PMID: 11058742

[ref2] Besada-Lombana P. B. Da Silva N. A. (2019). Engineering the early secretory pathway for increased protein secretion in *Saccharomyces cerevisiae*. Metab. Eng. 55, 142–151. doi: 10.1016/j.ymben.2019.06.010, PMID: 31220665

[ref3] Bishop R. E. (2000). The bacterial lipocalins. Biochim. Biophys. Acta 1482, 73–83. doi: 10.1016/s0167-4838(00)00138-2 11058749

[ref4] Choudhary V. Schneiter R. (2012). Pathogen-related yeast (PRY) proteins and members of the CAP superfamily are secreted sterol-binding proteins. Proc. Natl. Acad. Sci. U. S. A. 109, 16882–16887. doi: 10.1073/pnas.1209086109, PMID: 23027975 PMC3479496

[ref5] Chua G. L. Tan B. C. Loke R. Y. J. He M. Chin C. F. Wong B. H.. (2023). Mfsd2a utilizes a flippase mechanism to mediate omega-3 fatty acid lysolipid transport. Proc. Natl. Acad. Sci. U. S. A. 120:e2215290120. doi: 10.1073/pnas.2215290120, PMID: 36848557 PMC10013850

[ref6] Curry S. Mandelkow H. Brick P. Franks N. (1998). Crystal structure of human serum albumin complexed with fatty acid reveals an asymmetric distribution of binding sites. Nat. Struct. Biol. 5, 827–835. doi: 10.1038/1869, PMID: 9731778

[ref7] Darwiche R. El Atab O. Cottier S. Schneiter R. (2018). The function of yeast CAP family proteins in lipid export, mating, and pathogen defense. FEBS Lett. 592, 1304–1311. doi: 10.1002/1873-3468.12909, PMID: 29125629

[ref8] Darwiche R. Kelleher A. Hudspeth E. M. Schneiter R. Asojo O. A. (2016). Structural and functional characterization of the CAP domain of pathogen-related yeast 1 (Pry1) protein. Sci. Rep. 6:28838. doi: 10.1038/srep28838, PMID: 27344972 PMC4921858

[ref9] Darwiche R. Mène-Saffrané L. Gfeller D. Asojo O. A. Schneiter R. (2017). The pathogen-related yeast protein Pry1, a member of the CAP protein superfamily, is a fatty acid-binding protein. J. Biol. Chem. 292, 8304–8314. doi: 10.1074/jbc.M117.781880, PMID: 28365570 PMC5437237

[ref10] de Carvalho C. C. C. R. Caramujo M. J. (2018). The various roles of fatty acids. Molecules 23:2583. doi: 10.3390/molecules23102583, PMID: 30304860 PMC6222795

[ref11] Ertunc M. E. Sikkeland J. Fenaroli F. Griffiths G. Daniels M. P. Cao H.. (2015). Secretion of fatty acid binding protein aP2 from adipocytes through a nonclassical pathway in response to adipocyte lipase activity. J. Lipid Res. 56, 423–434. doi: 10.1194/jlr.M055798, PMID: 25535287 PMC4306695

[ref12] Esteves A. Ehrlich R. (2006). Invertebrate intracellular fatty acid binding proteins. Comp. Biochem. Physiol. C Toxicol. Pharmacol. 142, 262–274. doi: 10.1016/j.cbpc.2005.11.006 16423563

[ref13] Faergeman N. J. Black P. N. Zhao X. D. Knudsen J. DiRusso C. C. (2001). The acyl-CoA synthetases encoded within FAA1 and FAA4 in *Saccharomyces cerevisiae* function as components of the fatty acid transport system linking import, activation, and intracellular utilization. J. Biol. Chem. 276, 37051–37059. doi: 10.1074/jbc.M100884200, PMID: 11477098

[ref14] Fang Y. Tong G. C. Means G. E. (2006). Structural changes accompanying human serum albumin’s binding of fatty acids are concerted. Biochim. Biophys. Acta 1764, 285–291. doi: 10.1016/j.bbapap.2005.11.018, PMID: 16413837

[ref15] Ferreira R. Teixeira P. G. Siewers V. Nielsen J. (2018). Redirection of lipid flux toward phospholipids in yeast increases fatty acid turnover and secretion. Proc. Natl. Acad. Sci. U. S. A. 115, 1262–1267. doi: 10.1073/pnas.1715282115, PMID: 29358378 PMC5819412

[ref16] Flower D. R. North A. C. Sansom C. E. (2000). The lipocalin protein family: structural and sequence overview. Biochim. Biophys. Acta 1482, 9–24. doi: 10.1016/s0167-4838(00)00148-5, PMID: 11058743

[ref17] Ganfornina M. D. Åkerström B. Sanchez D. (2022). Editorial: functional profile of the Lipocalin protein family. Front. Physiol. 13:904702. doi: 10.3389/fphys.2022.904702, PMID: 35574442 PMC9096435

[ref18] Gietz R. D. Woods R. A. (2002). Screening for protein-protein interactions in the yeast two-hybrid system. Methods Mol. Biol. 185, 471–486. doi: 10.1385/1-59259-241-4:471 11769011

[ref19] Glasgow B. J. (2021). Tear Lipocalin and Lipocalin-interacting membrane receptor. Front. Physiol. 12:684211. doi: 10.3389/fphys.2021.684211, PMID: 34489718 PMC8417070

[ref20] Glatz J. F. Luiken J. J. Bonen A. (2010). Membrane fatty acid transporters as regulators of lipid metabolism: implications for metabolic disease. Physiol. Rev. 90, 367–417. doi: 10.1152/physrev.00003.2009 20086080

[ref21] Grabner G. F. Xie H. Schweiger M. Zechner R. (2021). Lipolysis: cellular mechanisms for lipid mobilization from fat stores. Nat. Metab. 3, 1445–1465. doi: 10.1038/s42255-021-00493-6, PMID: 34799702

[ref22] Han Z. Xiong D. Schneiter R. Tian C. (2023). The function of plant PR1 and other members of the CAP protein superfamily in plant-pathogen interactions. Mol. Plant Pathol. 24, 651–668. doi: 10.1111/mpp.13320, PMID: 36932700 PMC10189770

[ref23] Hasbargen K. B. Shen W. J. Zhang Y. Hou X. Wang W. Shuo Q.. (2020). Slc43a3 is a regulator of free fatty acid flux. J. Lipid Res. 61, 734–745. doi: 10.1194/jlr.RA119000294, PMID: 32217606 PMC7193958

[ref24] Henkin A. H. Ortegon A. M. Cho S. Shen W. J. Falcon A. Kraemer F. B.. (2012). Evidence for protein-mediated fatty acid efflux by adipocytes. Acta Physiol (Oxf.) 204, 562–570. doi: 10.1111/j.1748-1716.2011.02367.x, PMID: 21951599 PMC3271185

[ref25] Horvath A. Riezman H. (1994). Rapid protein extraction from *Saccharomyces cerevisiae*. Yeast 10, 1305–1310. doi: 10.1002/yea.320101007, PMID: 7900419

[ref26] Hotamisligil G. S. Bernlohr D. A. (2015). Metabolic functions of FABPs--mechanisms and therapeutic implications. Nat. Rev. Endocrinol. 11, 592–605. doi: 10.1038/nrendo.2015.122, PMID: 26260145 PMC4578711

[ref27] Iberg N. Flückiger R. (1986). Nonenzymatic glycosylation of albumin *in vivo*. Identification of multiple glycosylated sites. J. Biol. Chem. 261, 13542–13545. doi: 10.1016/S0021-9258(18)67052-8, PMID: 3759977

[ref28] Johnson D. R. Knoll L. J. Levin D. E. Gordon J. I. (1994). *Saccharomyces cerevisiae* contains four fatty acid activation (FAA) genes: an assessment of their role in regulating protein N-myristoylation and cellular lipid metabolism. J. Cell Biol. 127, 751–762. doi: 10.1083/jcb.127.3.751, PMID: 7962057 PMC2120220

[ref29] Josephrajan A. Hertzel A. V. Bohm E. K. McBurney M. W. Imai S. I. Mashek D. G.. (2019). Unconventional secretion of adipocyte fatty acid binding protein 4 is mediated by Autophagic proteins in a Sirtuin-1-dependent manner. Diabetes 68, 1767–1777. doi: 10.2337/db18-1367, PMID: 31171562 PMC6702637

[ref30] Kamp F. Hamilton J. A. (2006). How fatty acids of different chain length enter and leave cells by free diffusion. Prostaglandins Leukot. Essent. Fatty Acids 75, 149–159. doi: 10.1016/j.plefa.2006.05.003, PMID: 16829065

[ref31] Kampf J. P. Kleinfeld A. M. (2007). Is membrane transport of FFA mediated by lipid, protein, or both? An unknown protein mediates free fatty acid transport across the adipocyte plasma membrane. Physiology (Bethesda) 22, 7–14. doi: 10.1152/physiol.00011.2006 17289927

[ref32] Lapolla A. Fedele D. Reitano R. Aricò N. C. Seraglia R. Traldi P.. (2004). Enzymatic digestion and mass spectrometry in the study of advanced glycation end products/peptides. J. Am. Soc. Mass Spectrom. 15, 496–509. doi: 10.1016/j.jasms.2003.11.014, PMID: 15047055

[ref33] Leber C. Polson B. Fernandez-Moya R. Da Silva N. A. (2015). Overproduction and secretion of free fatty acids through disrupted neutral lipid recycle in *Saccharomyces cerevisiae*. Metab. Eng. 28, 54–62. doi: 10.1016/j.ymben.2014.11.006, PMID: 25461829

[ref34] Li F. Chen Y. Qi Q. Wang Y. Yuan L. Huang M.. (2022). Improving recombinant protein production by yeast through genome-scale modeling using proteome constraints. Nat. Commun. 13:2969. doi: 10.1038/s41467-022-30689-7, PMID: 35624178 PMC9142503

[ref35] Li N. Gügel I. L. Giavalisco P. Zeisler V. Schreiber L. Soll J.. (2015). FAX1, a novel membrane protein mediating plastid fatty acid export. PLoS Biol. 13:e1002053. doi: 10.1371/journal.pbio.1002053, PMID: 25646734 PMC4344464

[ref36] Lin Y. Feng Y. Zheng L. Zhao M. Huang M. (2023). Improved protein production in yeast using cell engineering with genes related to a key factor in the unfolded protein response. Metab. Eng. 77, 152–161. doi: 10.1016/j.ymben.2023.04.004, PMID: 37044356

[ref37] Liu Z. Wang J. Nielsen J. (2022). Yeast synthetic biology advances biofuel production. Curr. Opin. Microbiol. 65, 33–39. doi: 10.1016/j.mib.2021, PMID: 34739924

[ref38] Lu S. Zhou C. Guo X. Du Z. Cheng Y. Wang Z.. (2022). Enhancing fluxes through the mevalonate pathway in *Saccharomyces cerevisiae* by engineering the HMGR and β-alanine metabolism. Microb. Biotechnol. 15, 2292–2306. doi: 10.1111/1751-7915.14072, PMID: 35531990 PMC9328733

[ref39] Mioka T. Fujimura-Kamada K. Mizugaki N. Kishimoto T. Sano T. Nunome H.. (2018). Phospholipid flippases and Sfk1p, a novel regulator of phospholipid asymmetry, contribute to low permeability of the plasma membrane. Mol. Biol. Cell 29, 1203–1218. doi: 10.1091/mbc.E17-04-0217, PMID: 29540528 PMC5935070

[ref40] Morais Cabral J. H. Atkins G. L. Sánchez L. M. López-Boado Y. S. López-Otin C. Sawyer L. (1995). Arachidonic acid binds to apolipoprotein D: implications for the protein’s function. FEBS Lett. 366, 53–56. doi: 10.1016/0014-5793(95)00484-q, PMID: 7789516

[ref41] Morales-Kastresana A. Siegemund M. Haak S. Peper-Gabriel J. Neiens V. Rothe C. (2022). Anticalin®-based therapeutics: expanding new frontiers in drug development. Int. Rev. Cell Mol. Biol. 369, 89–106. doi: 10.1016/bs.ircmb.2022.03.009, PMID: 35777866

[ref42] Mori A. Hara S. Sugahara T. Kojima T. Iwasaki Y. Kawarasaki Y.. (2015). Signal peptide optimization tool for the secretion of recombinant protein from *Saccharomyces cerevisiae*. J. Biosci. Bioeng. 120, 518–525. doi: 10.1016/j.jbiosc.2015.03.003, PMID: 25912446

[ref43] Mumberg D. Muller R. Funk M. (1995). Yeast vectors for the controlled expression of heterologous proteins in different genetic backgrounds. Gene 156, 119–122. doi: 10.1016/0378-1119(95)00037-7, PMID: 7737504

[ref44] Payen C. Thompson D. (2019). The renaissance of yeasts as microbial factories in the modern age of biomanufacturing. Yeast 36, 685–700. doi: 10.1002/yea.3439, PMID: 31423599

[ref45] Pelosi P. Knoll W. (2022). Odorant-binding proteins of mammals. Biol. Rev. Camb. Philos. Soc. 97, 20–44. doi: 10.1111/brv.12787 34480392

[ref46] Prentice K. J. Saksi J. Hotamisligil G. S. (2019). Adipokine FABP4 integrates energy stores and counterregulatory metabolic responses. J. Lipid Res. 60, 734–740. doi: 10.1194/jlr.S091793, PMID: 30705117 PMC6446704

[ref47] Rassart E. Desmarais F. Najyb O. Bergeron K. F. Mounier C. (2020). Apolipoprotein D. Gene 756:144874. doi: 10.1016/j.gene.2020.144874, PMID: 32554047 PMC8011330

[ref48] Ren L. Yi J. Li W. Zheng X. Liu J. Wang J.. (2019). Apolipoproteins and cancer. Cancer Med. 8, 7032–7043. doi: 10.1002/cam4.2587, PMID: 31573738 PMC6853823

[ref49] Scharnewski M. Pongdontri P. Mora G. Hoppert M. Fulda M. (2008). Mutants of *Saccharomyces cerevisiae* deficient in acyl-CoA synthetases secrete fatty acids due to interrupted fatty acid recycling. FEBS J. 275, 2765–2778. doi: 10.1111/j.1742-4658.2008.06417.x, PMID: 18422644

[ref50] Schindelin J. Arganda-Carreras I. Frise E. Kaynig V. Longair M. Pietzsch T.. (2012). Fiji: an open-source platform for biological-image analysis. Nat. Methods 9, 676–682. doi: 10.1038/nmeth.2019, PMID: 22743772 PMC3855844

[ref51] Sheng J. Feng X. (2015). Metabolic engineering of yeast to produce fatty acid-derived biofuels: bottlenecks and solutions. Front. Microbiol. 6:554. doi: 10.3389/fmicb.2015.00554, PMID: 26106371 PMC4459083

[ref52] Sleep D. Belfield G. P. Goodey A. R. (1990). The secretion of human serum albumin from the yeast *Saccharomyces cerevisiae* using five different leader sequences. Biotechnology (N Y) 8, 42–46. doi: 10.1038/nbt0190-42, PMID: 1366511

[ref53] Son S. H. Kim J. E. Park G. Ko Y. J. Sung B. H. Seo J.. (2022). Metabolite trafficking enables membrane-impermeable-terpene secretion by yeast. Nat. Commun. 13:2605. doi: 10.1038/s41467-022-30312-9, PMID: 35546160 PMC9095633

[ref54] Stopková R. Otčenášková T. Matějková T. Kuntová B. Stopka P. (2021). Biological roles of Lipocalins in chemical communication, reproduction, and regulation of microbiota. Front. Physiol. 12:740006. doi: 10.3389/fphys.2021.740006, PMID: 34594242 PMC8476925

[ref55] Subramanian S. P. Gundry R. L. (2022). The known unknowns of apolipoprotein glycosylation in health and disease. iScience 25:105031. doi: 10.1016/j.isci.2022.105031, PMID: 36111253 PMC9468411

[ref56] Tegoni M. Pelosi P. Vincent F. Spinelli S. Campanacci V. Grolli S.. (2000). Mammalian odorant binding proteins. Biochim. Biophys. Acta 1482, 229–240. doi: 10.1016/s0167-4838(00)00167-9 11058764

[ref57] Teixeira P. G. Ferreira R. Zhou Y. J. Siewers V. Nielsen J. (2017). Dynamic regulation of fatty acid pools for improved production of fatty alcohols in *Saccharomyces cerevisiae*. Microb. Cell Factories 16:45. doi: 10.1186/s12934-017-0663-3, PMID: 28298234 PMC5353878

[ref58] Thompson B. R. Lobo S. Bernlohr D. A. (2010). Fatty acid flux in adipocytes: the in’s and out’s of fat cell lipid trafficking. Mol. Cell. Endocrinol. 318, 24–33. doi: 10.1016/j.mce.2009.08.015, PMID: 19720110 PMC2826553

[ref59] van den Berg B. Black P. N. Clemons W. M. Rapoport T. A. (2004). Crystal structure of the long-chain fatty acid transporter FadL. Science 304, 1506–1509. doi: 10.1126/science.1097524, PMID: 15178802

[ref60] Villeneuve J. Bassaganyas L. Lepreux S. Chiritoiu M. Costet P. Ripoche J.. (2018). Unconventional secretion of FABP4 by endosomes and secretory lysosomes. J. Cell Biol. 217, 649–665. doi: 10.1083/jcb.201705047, PMID: 29212659 PMC5800802

[ref61] Virtanen T. (2021). Inhalant mammal-derived Lipocalin allergens and the innate immunity. Front. Allergy 2:824736. doi: 10.3389/falgy.2021.824736, PMID: 35387007 PMC8974866

[ref62] Waters M. G. Evans E. A. Blobel G. (1988). Prepro-alpha-factor has a cleavable signal sequence. J. Biol. Chem. 263, 6209–6214. doi: 10.1016/S0021-9258(18)68773-3, PMID: 3283123

[ref9001] Xu W. Yuan J. Yang S. Ching C. B. Liu J. F. (2016). Programming saposin-mediated compensatory metabolic sinks for enhanced ubiquinone production. ACS Synth Biol. 5, 1404–1411. doi: 10.1021/acssynbio.6b00148, PMID: 27389347

[ref63] Yan Q. Pfleger B. F. (2019). Revisiting metabolic engineering strategies for microbial synthesis of oleochemicals. Metab. Eng. 58, 35–46. doi: 10.1016/j.ymben.2019.04.009, PMID: 31022535

[ref64] Yang H. H. Wang X. Li S. Liu Y. Akbar R. Fan G. C. (2023). Lipocalin family proteins and their diverse roles in cardiovascular disease. Pharmacol. Ther. 244:108385. doi: 10.1016/j.pharmthera.2023.108385, PMID: 36966973 PMC10079643

[ref65] Yu T. Zhou Y. J. Huang M. Liu Q. Pereira R. David F.. (2018). Reprogramming yeast metabolism from alcoholic fermentation to lipogenesis. Cells 174, 1549–1558.e14. doi: 10.1016/j.cell.2018.07.013, PMID: 30100189

[ref66] Zhou Y. J. Buijs N. A. Zhu Z. Qin J. Siewers V. Nielsen J. (2016). Production of fatty acid-derived oleochemicals and biofuels by synthetic yeast cell factories. Nat. Commun. 7:11709. doi: 10.1038/ncomms11709, PMID: 27222209 PMC4894961

[ref67] Zou Z. Tong F. Faergeman N. J. Borsting C. Black P. N. DiRusso C. C. (2003). Vectorial acylation in *Saccharomyces cerevisiae*. Fat1p and fatty acyl-CoA synthetase are interacting components of a fatty acid import complex. J. Biol. Chem. 278, 16414–16422. doi: 10.1074/jbc.M210557200, PMID: 12601005

