# Supplementary material for: Human lipocalins bind and export fatty acids through the secretory pathway of yeast cells
Source: Front Microbiol. 2024 Jan 5;14:1309024. doi: 10.3389/fmicb.2023.1309024 (PMC10849133; doi:10.3389/fmicb.2023.1309024)
Supplement: Supplementary file 1 [file Data_Sheet_1.PDF]

Supplementary Material

1 Supplementary Data

<u>Codon optimized sequences (CDS) of human lipid-binding proteins used in this study</u>

<u>Albumin CDS</u>

ATGGATGCTCATAAATCTGAAGTTGCACATAGATTCAAAGATTTGGGTGAAGAAAACTT
CAAGGCTTTGGTTTTGATCGCATTCGCTCAATATTTGCAACAATGTCCATTCGAAGATCA
TGTCAAGTTGGTTAACGAAGTTACTGAATTTGCTAAAACATGTGTTGCTGATGAATCTGC
AGAAAACTGTGATAAGTCATTGCATACTTTGTTCGGTGACAAATTGTGTACTGTTGCTAC
ATTAAGAGAAACATACGGTGAAATGGCTGATTGTTGTGCAAAGCAAGAACCAGAAAGA
AACGAATGTTTCTTGCAACATAAGGATGATAACCCAAATTTGCCAAGATTGGTTAGACC
AGAAGTTGATGTTATGTGTACTGCTTTTCATGATAACGAAGAAACATTTTTGAAGAAATA
TTTGTACGAAATCGCAAGAAGACATCCATACTTCTACGCTCCAGAATTGTTGTTTTTCGC
AAAGAGATACAAGGCTGCTTTTACTGAATGTTGTCAAGCTGCAGATAAAGCTGCATGTT
TGTTGCCAAAGTTGGATGAATTGAGAGATGAGGGTAAAGCTTCTTCAGCAAAACAAAGA
TTGAAGTGTGCTTCATTGCAAAAGTTCGGTGAAAGAGCTTTTAAAGCATGGGCTGTTGC
AAGATTGTCACAAAGATTTCCAAAGGCTGAATTCGCAGAAGTTTCTAAGTTGGTTACTG
ATTTGACAAAGGTTCATACAGAATGTTGTCATGGTGACTTGTTAGAATGTGCTGATGATA
GAGCTGATTTGGCAAAGTACATCTGTGAAAACCAAGATTCAATTTCTTCAAAATTAAAA
GAATGCTGTGAAAAGCCATTGTTGGAAAAATCTCATTGTATCGCTGAAGTTGAAAACGA
TGAAATGCCAGCAGATTTGCCATCTTTGGCTGCAGATTTCGTTGAATCAAAGGATGTTTG
TAAGAATTATGCTGAAGCAAAGGATGTTTTCTTGGGCATGTTCTTGTACGAATACGCTAG
AAGACATCCAGATTACTCTGTTGTTTTGTTGTTGAGATTGGCAAAGACTTACGAAACTAC
ATTGGAAAAGTGTTGTGCTGCAGCTGATCCACATGAATGTTACGCTAAGGTTTTCGATGA
ATTCAAACCATTGGTTGAAGAACCACAAAATTTGATTAAACAAAACTGTGAATTGTTTG
AACAATTGGGTGAATATAAATTTCAAAACGCTTTGTTGGTTAGATACACTAAGAAAGTT
CCACAAGTTTCTACTCCAACATTGGTTGAAGTTTCAAGAAATTTGGGTAAAGTTGGTTCT
AAATGTTGTAAACATCCAGAAGCTAAAAGAATGCCATGTGCAGAAGATTATTTGTCAGT
TGTTTTGAACCAATTGTGTGTTTTACATGAAAAGACTCCAGTTTCTGATAGAGTTACTAA
GTGTTGTACAGAATCATTGGTTAACAGAAGACCATGTTTCTCTGCTTTGGAAGTTGATGA
AACATACGTTCCAAAGGAATTCAATGCTGAAACTTTTACATTCCATGCAGATATCTGTAC
TTTGTCAGAAAAGGAAAGACAAATTAAGAAACAAACAGCTTTGGTTGAATTGGTTAAGC
ATAAGCCAAAGGCTACTAAGGAACAATTGAAGGCAGTTATGGATGATTTCGCAGCTTTC
GTTGAAAAATGTTGTAAGGCTGATGATAAGGAAACATGTTTTGCAGAAGAAGGTAAAA
AGTTAGTTGCAGCTTCTCAAGCAGCTTTAGGTTTGTGA

Fabp4 CDS

ATGTGTGATGCTTTTGTTGGTACTTGGAAGTTGGTTTCTTCAGAAAACTTCGATGATTAC
ATGAAAGAAGTTGGTGTTGGTTTTGCAACTAGAAAAGTTGCTGGTATGGCAAAGCCAAA
CATGATCATCTCAGTTAACGGTGACGTTATCACAATTAAATCTGAATCAACTTTTAAAAA
CACAGAAATCTCTTTTATTTTGGGTCAAGAATTCGATGAAGTTACAGCTGATGATAGAA
AGGTTAAGTCAACTATCACATTGGATGGTGGTGTTTTAGTTCATGTTCAAAAGTGGGATG
GTAAATCTACTACAATTAAAAGAAAGAGAGAAGATGATAAGTTGGTTGTTGAATGTGTT
ATGAAAGGTGTTACTTCTACAAGAGTTTACGAAAGAGCATGA

ApoD CDS

ATGGCTGAACAAGCCTTCCACTTGGGTAAATGTCCAAATCCACCAGTCCAAGAAAACTT
CGATGTAAACAAATACTTAGGTAGATGGTATGAAATCGAAAAGATTCCAACTACATTCG
AAAACGGTAGATGTATCCAAGCTAACTACTCTTTGATGGAAAATGGTAAAATTAAAGTT
TTGAACCAAGAATTGAGAGCAGATGGTACTGTTAATCAAATTGAAGGTGAAGCTACTCC
AGTTAATTTGACAGAACCAGCAAAGTTGGAAGTTAAATTTTCTTGGTTCATGCCATCAGC
TCCATACTGGATCTTGGCAACTGATTACGAAAACTACGCTTTGGTTTACTCATGTACATG
TATCATCCAATTGTTCCATGTTGATTTTGCTTGGATTTTGGCAAGAAATCCAAATTTGCC
ACCAGAAACTGTTGATTCTTTGAAAAATATTTTAACATCAAATAACATAGACGTAAAGA
AAATGACCGTAACAGACCAAGTAAATTGTCCTAAGTTGAGTTGA

Lcn1 CDS

ATGGCTGAACATCATTTGTTAGCTTCTGATGAAGAAATTCAAGATGTTTCAGGTACTTGG
TATTTGAAAGCAATGACAGTTGATAGAGAATTCCCAGAAATGAATTTGGAATCTGTTAC
TCCAATGACATTGACTACATTAGAAGGTGGTAATTTGGAAGCTAAGGTTACTATGTTGAT
CTCAGGTAGATGTCAAGAAGTTAAGGCAGTTTTGGAAAAGACTGATGAACCTGGTAAAT
ATACAGCTGATGGTGGTAAACATGTTGCATACATCATCAGATCTCATGTTAAGGATCATT
ACATTTTCTATTGTGAGGGTGAATTGCATGGTAAACCAGTTAGAGGTGTTAAGTTGGTTG
GTAGAGATCCTAAAAATAATTTGGAAGCTTTGGAAGATTTTGAAAAAGCTGCAGGTGCA
AGAGGTTTGTCTACTGAATCAATCTTGATCCCAAGACAATCAGAAACATGTTCTCCAGGT
TCAGATTGA

Obp2a CDS

ATGTTGTCTTTTACTTTGGAAGAAGAAGATATCACTGGTACATGGTATGTTAAGGCTATG
GTTGTTGATAAGGATTTCCCAGAAGATAGAAGACCAAGAAAAGTTTCACCAGTTAAAGT
TACAGCTTTGGGTGGTGGTAATTTGGAAGCAACTTTTACTTTTATGAGAGAAGATAGATG
TATCCAAAAGAAAATTTTGATGAGAAAAACTGAAGAACCTGGTAAATTTTCTGCTTACG
GTGGTAGAAAGTTGATATATTTGCAAGAATTGCCAGGTACAGATGATTACGTTTTCTATT

GTAAGGATCAAAGAAGAGGTGGTTTAAGATACATGGGTAAATTGGTTGGTAGAAACCC
AAACACTAATTTGGAAGCATTGGAAGAATTCAAGAAATTGGTTCAACATAAGGGTTTGT
CTGAAGAAGATATTTTTATGCCATTGCAAACAGGTTCATGTGTTTTGGAACATTGA
